# Amyloid Beta Oligomers Activate Death Receptors and Mitochondria-Mediated Apoptotic Pathways in Cerebral Vascular Smooth Muscle Cells; Protective Effects of Carbonic Anhydrase Inhibitors

**DOI:** 10.3390/cells12242840

**Published:** 2023-12-14

**Authors:** Amy Anzovino, Elisa Canepa, Micaelly Alves, Nicole L. Lemon, Roxana O. Carare, Silvia Fossati

**Affiliations:** 1 Alzheimer’s Center at Temple, Department of Neural Sciences, Lewis Katz School of Medicine, Temple University, 3500 N Broad St, Philadelphia, PA 19140, USA; ac.anzovino@gmail.com (A.A.); elisa.canepa@temple.edu (E.C.); micaelly.alves@temple.edu (M.A.); nicole.lemon@temple.edu (N.L.L.); 2 Faculty of Medicine, University of Southampton, Southampton SO16 6YD, UK; r.o.carare@soton.ac.uk

**Keywords:** amyloid beta, mitochondria, cerebral vascular smooth muscle cells, apoptosis, carbonic anhydrase

## Abstract

Amyloid beta (Aβ) deposition within the brain vasculature is an early hallmark of Alzheimer’s disease (AD), which triggers loss of brain vascular smooth muscle cells (BVSMCs) in cerebral arteries, via poorly understood mechanisms, altering cerebral blood flow, brain waste clearance, and promoting cognitive impairment. We have previously shown that, in brain endothelial cells (ECs), vasculotropic Aβ species induce apoptosis through death receptors (DRs) DR4 and DR5 and mitochondria-mediated mechanisms, while FDA-approved carbonic anhydrase inhibitors (CAIs) prevent mitochondria-mediated EC apoptosis in vitro and in vivo. In this study, we analyzed Aβ-induced extrinsic and intrinsic (DR- and mitochondria-mediated) apoptotic pathways in BVSMC, aiming to unveil new therapeutic targets to prevent BVSMC stress and death. We show that both apoptotic pathways are activated in BVSMCs by oligomeric Aβ42 and *Aβ40-Q22 (AβQ22)* and mitochondrial respiration is severely impaired. Importantly, the CAIs methazolamide (MTZ) and acetazolamide (ATZ) prevent the pro-apoptotic effects in BVSMCs, while reducing caspase 3 activation and Aβ deposition in the arterial walls of TgSwDI animals, a murine model of cerebral amyloid angiopathy (CAA). This study reveals new molecular targets and a promising therapeutic strategy against BVSMC dysfunction in AD, CAA, and ARIA (amyloid-related imaging abnormalities) complications of recently FDA-approved anti-Aβ antibodies.

## 1. Introduction

Cerebrovascular dysfunction and the build-up of amyloid beta (Aβ) in the walls of cerebral arterioles, known as cerebral amyloid angiopathy (CAA), represent early key elements of Alzheimer’s disease (AD). The accumulation of Aβ leads to further loss of brain vascular smooth muscle cells (BVSMCs) in cerebral arteries via poorly known mechanisms, promoting cerebral blood flow alterations, worsening clearance mechanisms, and contributing to cognitive impairment [1,2,3,4,5]. Various Aβ species are deposited within cerebral arterioles and capillaries in AD and CAA, leading to detrimental effects on BVSMCs [6]. Among these, Aβ42, due to its high propensity to quickly form aggregates, is known as the most toxic isoform. Our lab and others have shown that the familial Aβ variant AβQ22 (the Dutch mutant), associated with an aggressive cerebrovascular clinical phenotype with severe CAA in humans, induces pro-apoptotic pathways in cerebral vascular cells [7,8]. By comparison, Aβ40-WT, although more abundant than Aβ42 in vascular deposits, is less cytotoxic to BVSMCs in vitro, with apoptosis occurring after several days of exposure [7]. We have previously demonstrated that in human brain microvascular endothelial cells, Aβ vasculotropic peptides trigger apoptosis by directly binding to and activating the TNF-related apoptosis-inducing ligand (TRAIL) death receptors (DRs) DR4 and DR5 [8].

Sporadic non-familial CAA is likely due to a failure of clearance of Aβ rather than overproduction [4,9]. Aβ is eliminated along the basement membranes of capillaries and basement membranes surrounding BVSMCs as intramural periarterial drainage (IPAD) [5]. The spontaneous contractions of BVSMCs represent the motive force for IPAD, failing in aging [10,11] and resulting in accumulation of Aβ in the walls of cerebral capillaries and arteries, causing or worsening CAA [9].

Although the degeneration of BVSMCs in Aβ-laden vessels has been previously shown, little progress has been made to define the specific cell death mechanisms activated by Aβ in these mural cells, which are extremely important in maintaining cerebral blood flow and clearance of deleterious material from the brain. Defining specific molecular mechanisms is essential to discover possible therapeutic targets to limit the toxic effects of Aβ on the brain vasculature, which are causal and early determinants of AD and CAA cognitive dysfunction. Targeting these pathways may also help to reduce the development of ARIA (amyloid-related imaging abnormalities) [4,12], an important side effect of the novel FDA-approved monoclonal anti-Aβ antibodies, used as current AD-modifying therapy.

Apoptotic cell death can occur through two distinct pathways: intrinsic and extrinsic. The extrinsic pathway begins with the binding of an extracellular ligand that activates a DR [13,14]. Once activated, these transmembrane receptors recruit the intracellular adaptor molecule FADD (Fas-associated protein with death domain), leading to the formation of DISC (death-inducing signaling complex) and cleavage of procaspase 8 to active caspase 8 to initiate apoptosis. In this pathway, caspase signaling is initiated through caspase 8 activation, which then cleaves BID (BH3-interacting domain death agonist) to truncated BID (tBID); tBid translocates to the mitochondria and engages the intrinsic, mitochondria-mediated apoptotic pathway by disinhibiting pro-apoptotic proteins such as BAX or inhibiting anti-apoptotic Bcl2 family members. This results in mitochondrial outer membrane permeabilization (MOMP), cytochrome C (CytC) release, and ultimately the activation of caspase 9 [8,15]. The intrinsic apoptotic pathway can also be independently activated by intracellular signals such as direct DNA damage, oxidative stress, and others that trigger MOMP [16,17]. MOMP results in the release of cell death modulators, such as CytC or apoptosis-inducing factor (AIF), from the mitochondrial intermembrane space [18,19,20] and can be associated with a reduction in mitochondrial ATP synthesis [21], inhibition of the respiratory chain, and increased reactive oxygen species (ROS) production [22]. CytC release induces the activation of caspase 9 through the formation of the apoptosome. The converging downstream cell death effector of both caspases 8 and 9 is caspase 3.

The aim of this study was to investigate the potential involvement of DR- and mitochondria-mediated pathways in the execution of Aβ oligomer-induced apoptosis in BVSMC and to test a new possible therapeutic avenue to prevent these effects. Overall, these investigations may aid in achieving a therapeutic strategy to diminish or prevent cerebrovascular compromise and clearance impairments in AD and CAA, as well as in discovering possible new targets and interventions against ARIA.

Our lab has demonstrated that inhibiting a class of enzymes known as carbonic anhydrases (CAs) rescued Aβ-mediated apoptosis in cerebral microvascular endothelial cells by preventing mitochondria-mediated pro-apoptotic mechanisms, such as CytC release, loss of mitochondrial membrane potential, production of mitochondrial ROS, and others [7,23,24,25,26]. CAs have many essential physiological functions, including maintaining pH and ion homeostasis, metabolism, and regulating cerebral blood flow [27,28]. This family of zinc metalloenzymes catalyzes the reversible hydration of carbon dioxide to produce bicarbonate and a proton (CO_2_ + H_2_O ←→ HCO_3_^−^ + H^+^) [29,30]. Studies have shown that CAs have potential as a therapeutic target in peripheral and central nervous system disorders, and we were the first to propose that they may be involved in AD pathology [23,24,25,26,28,29,30,31]. In our recent in vivo studies, the FDA-approved CA inhibitors (CAIs) methazolamide (MTZ) and acetazolamide (ATZ) have been shown to protect against cerebrovascular pathology, glial overactivation, and cognitive dysfunction in a mouse model of AD and CAA [24]. However, very little is known about the effects of these compounds in modulating the effects of Aβ on BVSMC health.

The present study characterizes specific and independent DR4/5- and mitochondria-mediated apoptotic pathways, which ultimately converge in BVSMC dysfunction and death in AD and CAA. Moreover, we show that CAIs prevent the toxic effects of Aβ peptides on these apoptotic mechanisms in BVSMCs.

## 2. Materials and Methods

### 2.1. Aβ Preparation and Oligomerization

AβQ22 and Aβ42 peptides were synthesized from Peptide 2.0 (Chantilly, WV, USA). The peptides were weighed, dissolved (1 mM) with HFIP, incubated at room temperature overnight, and then lyophilized. HFIP-pre-treated and lyophilized peptides were then resuspended at 10 mM in DMSO, as we previously described [32]. For oligomeric preparations, lyophilized peptides at 10 mM in DMSO were then diluted to 100 µM in serum-free media and incubated at 4 °C overnight to aggregate, following established protocols [33]. Preformed oligomers were then added to treatment media at the desired final concentrations, either alone or in the presence of CAIs.

### 2.2. Cell Culture

Primary human BVSMCs (ScienCell, San Diego, CA, USA) were grown in SMC medium containing 2% FBS, SMC growth supplement (ScienCell), and penicillin/streptomycin, as recommended by the manufacturer. Cells were maintained at 37 °C with 5% CO_2_. For treatment, cells were switched to serum-free SMC medium with penicillin/streptomycin but no growth supplements.

### 2.3. Methazolamide and Acetazolamide Preparation

The CAIs methazolamide (MTZ) and acetazolamide (ATZ) were dissolved in DMSO to make 100 mM stock solutions, then diluted to the final concentrations in the serum-free medium used for each experiment.

### 2.4. Cell Death ELISA

BVSMCs were seeded in 24 well plates (20,000 cells/well) and left to attach overnight at 37 °C with 5% CO_2_. The next day, cells were treated with 10 μM Aβ oligomers. Apoptosis was assessed by quantitating fragmented nucleosome formation using Cell death ELISA^PLUS^ (Roche Applied Science, Penzberg, Germany), as recommended by the manufacturer.

### 2.5. Cell Event Fluorescent Caspase 3/7 Assay

Cleaved (active) Caspase 3/7 was measured using the CellEvent^TM^ Caspase-3/7 Green Detection Reagent (ThermoFischer Scientific, Waltham, MA, USA). A total of 10,000 cells/well were seeded in a 96-well plate and were treated for 6 or 24 h with the Aβ oligomers. Following treatment, the media was removed, and the fluorescent Caspase 3/7 Green Detection Reagent was added (5 μM stock concentration). Cells were incubated with the dye for 1 h at 37 °C. Nuclei were stained with Hoechst. Cells were imaged using an EVOS M5000 microscope (Carl Zeiss, Oberkochen, Germany).

### 2.6. Mitochondrial Stress Test

BVSMCs were plated in a 96-well Seahorse microplate at 20,000 cells/well and left to attach overnight at 37 °C with 5% CO_2_. The next day, cells were treated with 10 μM Aβ oligomers, in the presence or absence of 100 μM ATZ or 300 μM MTZ, and incubated for 24 h at 37 °C with 5% CO_2_. After 24 h, the Seahorse Analyzer XF96 was used to measure multiple parameters of mitochondrial respiration (basal respiration, ATP-linked production, maximal respiration, and spare respiratory capacity), following 3 serial injections of oligomycin, FCCP, and rotenone + antimycin A (final concentrations: 1.5 μM oligomycin, 1.5 μM FCCP, and 0.5 μM rotenone and antimycin A). FCCP concentrations were titrated from 1.0 μM to 2.5 μM and cell numbers were optimized as recommended by the manufacturer (Agilent, Santa Clara, CA, USA), before performing the final experiments. Measurements were normalized to protein concentration (μg/μL).

### 2.7. Western Blot

Proteins were separated on 4–20% Tris–glycine gradient gels (Invitrogen, Waltham, MA, USA) by electrophoresis. Proteins were transferred onto PVDF membranes (BIORAD, Hercules, CA, USA) at 24 V for 16 h at 4 °C. Membranes were then blocked for 1 h at room temperature (RT) in 5% non-fat milk in Tris-buffered saline with 0.1% Tween-20 (TBST). Membranes were blotted with primary antibodies against DR4 (Invitrogen, Waltham, MA, USA), DR5 (ThermoFisher, Waltham, MA, USA), cleaved caspase 8 (Novus, Centennial, CO, USA), Bax (Novus), BID and tBID (Cell Signaling, Denver, CO, USA), FLIP (Cell Signaling), Bcl2 (Abcam, Cambridge, UK), and actin (EMD Millipore, Burlington, VT, USA), diluted in 3% BSA/TBST for 1 h at RT. Following incubation with primary antibodies, membranes were incubated with anti-rabbit or anti-mouse IRDye-conjugated secondary antibodies diluted 1:15,000 in 5%BSA/TBST for 1 h at RT. Membranes were imaged with LI-COR ODYSSEY.

### 2.8. Quantitative PCR

RNA was isolated using the Qiagen RNeasy kit (Qiagen #74104, Germantown, MD, USA), reverse transcribed using the Super Script cDNA synthesis kit (11754050), and then evaluated for expression of *TNFRS10A*, *TNFRS10B,* and *Cyclophilin B* using Taqman Gene Expression Assays (Taqman Primers from ThermoFisher: TNFRSF10A (DR4) #HS00269492; TNFRSF10B (DR5) #HS00366272; CYC B #HS00168719); qPCR was performed using the QuantStudio 6 Pro Real-Time PCR system from Applied Biosystems.

### 2.9. Immunocytochemistry

BVSMCs were plated on 12 mm sterile glass coverslips coated with cell attachment factor (Cell Systems) at 70% confluence. After 24 h, cells were treated with Aβ oligomers for 6 or 24 h. Cells were then washed with 1x PBS, fixed with 3.7% paraformaldehyde (15′, RT), washed again, permeabilized with 0.2% Triton (TX100) in PBS (10′, RT), and blocked with 3% BSA in PBS (1 h, RT). Coverslips were then incubated with mouse monoclonal anti-DR4, anti-Cytochrome C conjugated to Alexa Fluor 488, and goat polyclonal anti-DR5, for 1 h at RT. Coverslips were washed 3x with 1x PBS for 5′ and then incubated with the species-appropriate secondary antibodies for 1 h at RT in the dark. Coverslips were then washed and mounted to slides with mounting media plus DAPI. Images were acquired with a Nikon Ti2-E fluorescence 216 deconvolution microscope equipped with 340/380, 465/495, 540/580, and 590/650 nm excitation 217 filters, with a 40x oil immersion objective lens.

### 2.10. Caspase 9 Activity Assay

Caspase 9 activity was measured using luminescent assays (Caspase-Glo 9, Promega, Madison, WI, USA). A total of 10,000 cells/well were plated in 96-well plates and incubated at 37 °C with 5% CO_2,_ with AβQ22 or Aβ42 oligomers for 2 to 24 h, in the presence or absence of CAIs. Cells were then incubated with Caspase-Glo reagent for 40′ at RT in the dark. The proteasome inhibitor MG-132 was added to the Caspase-Glo reagent to inhibit non-specific background activity. After 40′, cell lysates were transferred to an opaque 96-well plate, and luminescence was measured in a plate-reading luminometer (SpectraMax 3). Results are expressed as fold change compared to untreated control cells.

### 2.11. DR4 and DR5 Gene Silencing

*TNFRSF10A* (siRNA ID#s16764) and *TNFRSF10B* (siRNA ID#s16756) were silenced by transient transfection with Silencer Select siRNA (Ambion, ThermoFisher, Waltham, MA, USA) using Lipofectamine RNAimax (ThermoFisher) diluted in Opti-MEM media (Gibco, ThermoFisher, Watham, MA, USA). Cells were incubated with *TNFRSF10A, TNFRSF10B,* or Silencer Select negative control (sc-siRNA) for 24 h. Transfection media was then removed and cells were treated with 10 µM oligomeric AβQ22 or Aβ42 for 24 h. Gene expression was assessed by qPCR at the same time point.

### 2.12. Mitotracker CMHX2ROS

250,000 BVSMCs were seeded on coverslips and treated with 10 µM oligomeric AβQ22 or Aβ42 (with or without CAIs) for 6 h. At the end of the treatment, conditioned media were removed, cells were washed with 1xPBS and then incubated for 45′ with 500 nm Mitotracker CMHX2ROS (ThermoFisher, Waltham, MA, USA), at 37 °C with 5% CO_2_, to visualize healthy mitochondria (this dye is internalized in mitochondria upon a healthy membrane potential). Media was removed after incubation and cells were washed with 1X PBS, prior to fixation with 3.7% paraformaldehyde, 15 min at 37 °C. Coverslips were mounted onto slides with DAPI fluoromount-G mounting medium and left to dry overnight in the dark. Images were acquired with a Nikon Ti2-E microscope with 60 and 100X oil immersion objective lenses. Quantification of the number of healthy mitochondrial particles in each cell was performed by ImageJ, establishing a threshold within the cell image to select only clearly defined particles, corresponding to the mitochondria presenting a healthy membrane potential (N = at least 5 images/group).

### 2.13. TgSwDI Animals

TgSwDI (Swedish, Dutch, IA, USA) mice (C57BL6/6 background) were kindly provided by Dr. Thomas Wisniewski (New York University, NYU, New York, NY, USA) and bred internally. They express the human APP gene (isoform 770) carrying the Swedish (K670N/M671L), Dutch (E693Q), and Iowa (D694N) mutations, under the control of the mouse neuronal Thy1 promoter, which induce extensive Aβ overproduction and deposition, mostly around the brain vasculature (CAA). The animals were housed in accordance with Institutional and NIH guidelines, and the animal protocol was approved by the IACUC Committee. Mice were maintained under controlled conditions (at ~22 °C, with an inverted 12 h light/dark cycle), with ad libitum access to food and water.

### 2.14. TgSwDI Treatment with CAIs

In order to evaluate the effects of ATZ and MTZ on Aβ-mediated cell death mechanisms in TgSwDI mice, we fed animals for 8 months (from 8 to 16 months of age) with MTZ- or ATZ-supplemented chow and compared them to TgSwDI mice fed with a control diet, as we recently published [24].

CAIs were incorporated into the rodent diet at 100 ppm (5053 by TestDiets, Quakertown, PA, USA).

### 2.15. Immunohistochemistry in TgSwDI

For immunostaining evaluation, 16-month-old brain sections were blocked with 10% NGS and 1% BSA solution for 2 h at RT and then incubated overnight at 4 °C with primary antibodies diluted in 0.1% Triton X-100 (Sigma, St. Louis, USA) blocking solution. Slices were stained with the following primary antibodies: Ms anti-Aβ (BioLegend, San Diego, CA, USA -clone 6E10, 1:500) and Rb anti-cleaved (active) caspase-3 (Cell Signaling, Danvers, CO, USA, 1:500). The following day, the species-appropriate Alexa Fluor-conjugated secondary antibodies (ThermoFisher, Waltham, MA, USA, 1:1000) and the 647-conjugated primary antibody anti-Smooth Muscle Actin (SMA, St Cruz) were employed (2 h at RT), following which, 1.5 μg/mL DAPI (Invitrogen, Waltham, MA, USA -D21490) was used as nuclear staining (10′ at RT). Stained sections were imaged with a Nikon Ti2-E fluorescence deconvolution microscope equipped with 340/380, 465/495, 540/580, and 590/650 nm excitation filters, using either 60× or 100× zoom objectives. Using a 0.5 μm Z-stack, 60× or 100× images were acquired. For consistent examination, 2 or 3 different images for each animal were acquired in the same brain area of interest.

## 3. Results

### 3.1. Prolonged Challenge with Aβ42 and AβQ22 Oligomers Induces Apoptosis in Human Brain Vascular Smooth Muscle Cells

**Figure 1:**
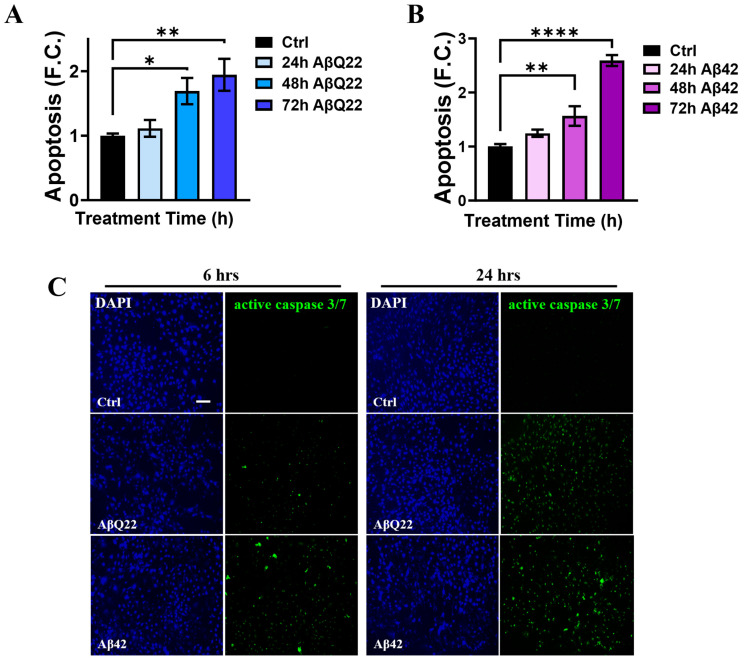
**Time course of Aβ oligomer-induced apoptosis in BVSMCs.** In (**A**,**B**), apoptosis is measured as DNA fragmentation in BVSMCs, following a time course after Aβ treatment. Both 10 μM AβQ22 (**A**) and Aβ42 (**B**) oligomers significantly prompted cell death in BVSMC after 48 and 72 h, with no cell death observed at 24 h. (**C**) Representative IF images, showing caspase 3 and 7 activation (green) in live BVSMCs, detected as early as 6 h, following both 10 μM oligomeric AβQ22 and Aβ42 challenge. Caspase 3 activation persisted and was exacerbated at a later time point (24 h). Cell nuclei were stained with DAPI (blue). Original magnification,10x. Scale bar, 100 μm. Data and images represent the combination of at least 3 independent experiments, each with 2 replicates. For (**A**,**B**), data are plotted as mean + SEM, and expressed as fold change (F.C.), compared to untreated cells (Ctrl). Statistical significance was evaluated by one-way ANOVA followed by Dunnett’s multiple comparison test. * = *p* < 0.05, ** = *p* < 0.01, and **** = *p* < 0.001, compared to untreated BVSMCs.

The pro-apoptotic effects of Aβ in cerebral vascular cells differ depending on cell type, Aβ species, aggregation state, and time of exposure [7,8]. Since oligomers are considered the most toxic species of Aβ for many cell types, including vascular cells [7,34,35,36,37,38], we aimed to elucidate the apoptotic effects of Aβ42 and AβQ22 oligomers in BVSMCs. AβQ22 and Aβ42 are among the most toxic peptides for the cerebral vasculature, as they aggregate with faster kinetics compared to Aβ40-WT and induce a more dramatic toxicity in cerebrovascular cells [36]. Here, human BVSMCs were treated with oligomeric Aβ42 or AβQ22 for 24, 48, and 72 h, at a concentration (10 µM) which was also shown in our previous studies to induce apoptosis in cerebral endothelial cells [32], and fragmented nucleosome generation was evaluated by cell death ELISA^PLUS^ (Figure 1A,B). Compared to untreated control cells, both AβQ22 (Figure 1A) and Aβ42 (Figure 1B) oligomers induced significant apoptosis (≥1.5-fold change, F.C.) after 48 h exposure, which was further increased after 72 h (≥2 F.C.). To ascertain the timing of cell death initiation induced by Aβ, we measured the activation of the executioner caspases 3/7 in live BVSMCs treated with Aβ42 or AβQ22 for 6 and 24 h. A modest increase in caspase 3/7 activation was detected after 6 h of treatment with either Aβ peptide (Figure 1C). At 24 h, both Aβ species induced a substantial activation of caspase 3/7 relative to untreated cells (Figure 1C). These results show that oligomeric AβQ22 and Aβ42 cause caspase-mediated apoptosis to a similar level in BVSMCs. Interestingly, we observed that an early modest activation of caspase 3/7 occurs at 6 h following both Aβ peptide treatments, with a substantial increase at 24 h.

### 3.2. Aβ Oligomers Impair Mitochondrial Respiration in BVSMCs

**Figure 2:**
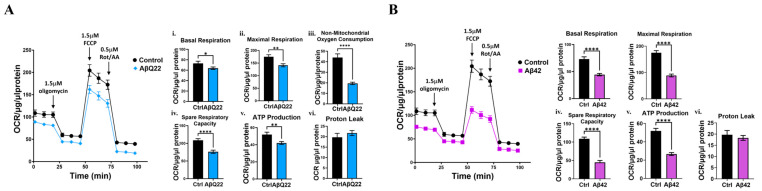
**Aβ oligomers impair mitochondrial respiration in BVSMCs.** (**A**,**B**) Measurement of mitochondrial respiration in BVSMCs, treated for 24 h with 10 μM AβQ22 (**A**) and Aβ42 (**B**). Both AβQ22 (**A**) and Aβ42 (**B**) significantly impaired oxygen consumption rate (OCR) basal respiration (**i**), maximal respiration (**ii**)**,** non-mitochondrial oxygen consumption (**iii**)**,** spare respiratory capacity (**iv**), and ATP production (**v**) in BVSMCs, without affecting proton leak (**vi**). Measurements were plotted following normalization to protein concentration. Graphs represent 3 independent experiments, with at least 6 replicates per group. Data are plotted as mean + SEM and statistical significance was evaluated by unpaired *t*-test. * = *p* < 0.05, ** = *p* < 0.01 and **** = *p* < 0.0001, compared to untreated BVSMCs.

Given that Aβ42 and AβQ22 oligomers engage apoptotic pathways with overt cell death starting at 48 h, as indicated by DNA fragmentation (Figure 1A,B), we sought to determine whether Aβ challenge induced an impairment in mitochondrial respiratory capacity at times preceding the execution of apoptosis, but at which the cells exhibited signs of stress, such as caspase activation. Therefore, we chose to test mitochondrial respiratory stress at 24 h, using the same concentration of oligomers as above. Using the Seahorse extracellular flux analyzer, we observed that AβQ22 caused a significant reduction in mitochondrial oxygen consumption rates (OCRs) after 24 h, relative to untreated cells (Figure 2A), with a significant decrease in basal respiration, maximal respiration, non-mitochondrial oxygen consumption, spare respiratory capacity, and ATP production, relative to untreated BVSMCs. Aβ42 oligomers even more dramatically reduced basal respiration, maximal respiration, non-mitochondrial oxygen consumption, spare respiratory capacity, and ATP production, all to approximately 50% OCR, compared to untreated cells (Figure 2B). These data show that both AβQ22 and Aβ42 oligomers cause mitochondrial metabolic dysfunction in BVSMCs, with Aβ42 challenge demonstrating a greater impact on mitochondrial respiration than AβQ22 at this time point (24 h). These results suggest that a failure of mitochondrial metabolic function precedes apoptosis in BVSMCs exposed to Aβ. This metabolic inefficiency may contribute to perivascular clearance impairments in AD and CAA brains.

### 3.3. DR4 and DR5 Mediate Aβ Oligomers’ Toxicity in BVSMCs

**Figure 3:**
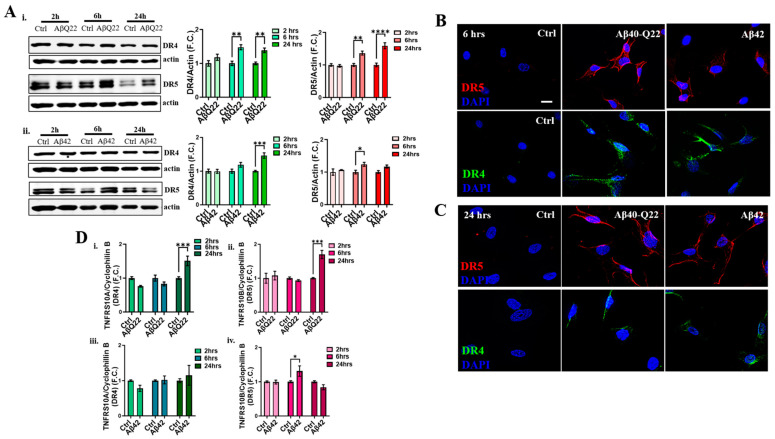
**AβQ22 and Aβ42 upregulate DR4 and DR5 expression in BVSMCs.** (**A**) Immunoblot analysis of death receptors 4 and 5 (DR4 and DR5) in BVSMCs treated with 10 μM AβQ22 (**i**) and Aβ42 (**ii**) for 2, 6 and 24 h. Six- and twenty-four-hour AβQ22 treatment significantly induced both DR4 and DR5 expression in BVSMCs (**i**), compared to untreated cells, while Aβ42 preferentially triggered an early DR5 (6 h) and a late DR4 (24 h) upregulation (**ii**). Graphs show F.C. of protein expression normalized to actin, and compared to untreated cells. (**B**,**C**) Representative ICC images demonstrate DR4 and DR5 increased expression, as well as a membranal localization in BVSMCs after 6 (**B**) and 24 h (**C**) treatment with AβQ22 and Aβ42. Cell nuclei are stained with DAPI (blue). Original magnification, 60x. Scale bar, 25 μm. (**D**) The evaluation of DR gene expression by RT-PCR demonstrated that AβQ22 significantly increases both DR4 (**i**) and DR5 (**ii**) mRNA expression at 24 h. Conversely, Aβ42, significantly induces DR5 (**iv**) mRNA upregulation as early as 6 h, without affecting DR4 (**iii**) transcript levels. Plots show F.C. of mRNA expression normalized to Cyclophilin B, and compared to untreated Ctrl BVSMCs. For (**A**–**D**), data and images represent the combination of 3 independent experiments, each with at least 2 replicates. For (**A**) and (**D**), data are plotted as mean + SEM and statistical significance was evaluated by two-way ANOVA followed by Šídák’s multiple comparisons test. * = *p* < 0.05, ** = *p* < 0.01, *** = *p* < 0.001, and **** = *p* < 0.0001, compared to relative Ctrl.

Given that our group has previously shown that Aβ upregulates and activates DR4 and DR5 in cerebral microvascular endothelial cells, leading to activation of the extrinsic apoptotic pathway, we hypothesized that this pathway may also be one of the initiators of Aβ-mediated apoptosis in BVSMCs. The extrinsic apoptotic pathway, initiated by the activation of cell-membrane death receptors, including the TRAIL DRs DR4 and DR5 [13], also leads to the engagement of the intrinsic apoptotic pathway, resulting in mitochondrial dysfunction and CytC release. We have demonstrated that Aβ oligomers function as alternative ligands for the TRAIL DRs, specifically through the binding of oligomeric species to the extracellular portion of DR4 and DR5 in solution [8]. Hence, we asked whether these DRs were also involved in the apoptotic events triggered by Aβ42 and AβQ22 oligomers in BVSMCs. Because DR activation is typically accompanied by their upregulation [8,39,40,41,42], DR4 and DR5 expression was probed by WB after BVSMCs were treated with Aβ42 or AβQ22 for 2, 6, and 24 h. Pathological upregulation of both DR4 and DR5 occurred as soon as 6 h, persisting at 24 h when BVSMCs were challenged with AβQ22 oligomers (Figure 3A(i)). By comparison, DR5 expression was significantly increased by Aβ42 treatment at 6 h, while DR4 expression was induced at a later time point (24 h) (Figure 3A(ii)). ICC analysis indicated that DR4 and DR5 expression on BVSMC membranes was increased after 6 and 24 h of treatment with both Aβ42 and AβQ22 (Figure 3B,C). To examine whether the increase in DR expression could be attributed to changes in their transcription, BVSMCs were treated for 2, 6, and 24 h with Aβ42 or AβQ22, and DR4 and DR5 gene expression was measured by qPCR. AβQ22 significantly upregulated *TNFRSF10A* (DR4) and *TNFRSF10B* (DR5) mRNA expression after 24 h of treatment (Figure 3D(i,ii)). Conversely, Aβ42 induced *TNFRSF10B* gene transcription at 6 h, with no significant changes in *TNFRSF10A* expression over time (Figure 3D(iii,iv)). Together, these results show that Aβ42 and AβQ22 oligomers trigger the pathological expression of DR4 and DR5, although with slightly different timelines, suggesting that DR4 and DR5 activation may be one of the main events leading to Aβ-oligomer mediated apoptosis in BVSMCs. These data also indicate a preferential transcriptional upregulation of DR5 by Aβ42, while AβQ22 affects the transcription of both receptors.

**Figure 4:**
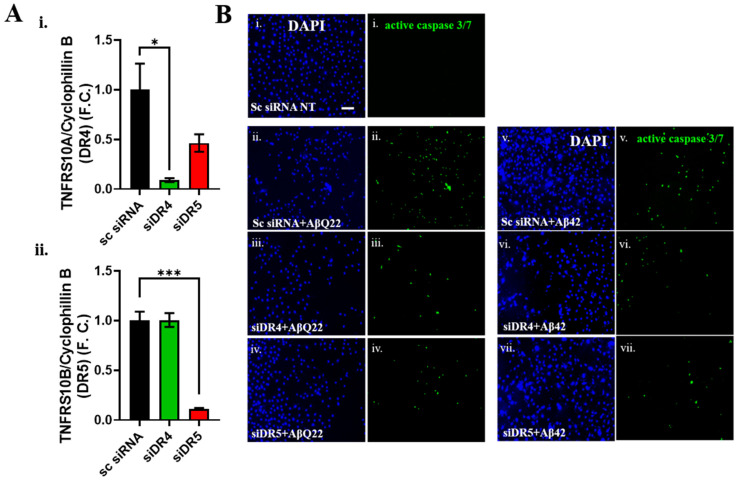
**DR4 and DR5 silencing protects BVSMCs from apoptosis.** (**A**) RT-PCR confirmed the efficiency of DR4 and DR5 transcript silencing in BVSMCs: *TNFRS10A* (**i**) and *TNFRS10B* (**ii**) mRNA levels were significantly reduced (>80%), compared to Scrambled (Sc) siRNA-treated BVSMCs (control cells). Data are plotted as F.C., compared to control BVSMCs and expressed as mean + SEM. Statistical significance was evaluated by Ordinary one-way ANOVA, followed by Dunnett’s multiple comparisons test. * = *p* < 0.05, and *** = *p* < 0.001, compared to untreated BVSMCs. (**B**) Assessment of active caspase 3 and 7 fluorescence (green), following 10 μM Aβ treatment in the presence or absence of DR knockdown, in BVSMCs. Compared to control BVSMCs (**i**) (scrambled silencing RNA: Sc siRNA, Aβ-non-treated: NT), AβQ22 (**ii**) induced caspase 3 activation at 24 h, which was rescued by both DR4 (**iii**) and DR5 (**iv**) silencing. Similarly, Aβ42 (**v**) activated caspase 3, and DR4 (**vi**) and DR5 (**vii**) knockdown attenuated it. Cell nuclei are stained with DAPI (blue). Original magnification, 10×. Scale bar, 100 μm.

Based on the above findings, DR4/5 may be important mediators of Aβ-induced BVSMC death. To corroborate this hypothesis, and demonstrate the causal effects of DR4/5 activation on BVSMCs apoptosis, we knocked down DR4 and DR5 by siRNA (Figure 4A). We confirmed the efficiency of DR4 and DR5 silencing (>80% *TNFRS10A* (DR4) and *TNFRS10B* (DR5) transcript level reduction with the respective siRNAs, compared to a scrambled (Sc) siRNA, Figure 4A(i,ii)). We then challenged the BVSMCs with AβQ22 and Aβ42 oligomers after DR knockdown, compared to Sc siRNA, and assessed caspase 3 activation (Figure 4B). Importantly, we observed that knockdown of either DR4 or DR5 prevented the Aβ-induced activation of caspase 3 after 24 h Aβ challenge (Figure 4B), demonstrating for the first time that DR4 and DR5 are causal mediators of Aβ-induced apoptosis in BVSMCs.

### 3.4. Aβ Oligomers Induce Caspase 8 Activation, CytC Release from the Mitochondria, and Caspase 9 Activation in Human BVSMCs

**Figure 5:**
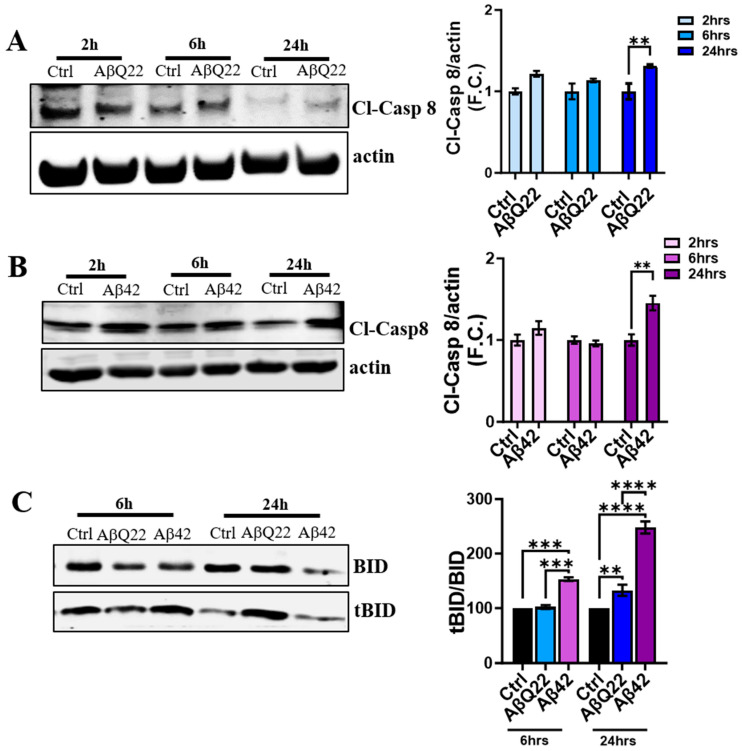
**Aβ activates caspase 8 in BVSMCs.** (**A**) and (**B**), WB analysis demonstrated that caspase 8 is active 24 h after 10 μM AβQ22 (**A**) and Aβ42 (**B**) treatments, shown as increased cleaved caspase 8 (CL-Casp8) expression in BVSMCs, while no significant changes were observed at earlier time points (2 and 6 h) in both Aβ species treatments. Graphs show F.C. of Cl-Casp8 normalized to actin, and compared to Ctrl. (**C**) At 6 h, Aβ42 led to an increased ratio of tBID/BID (truncated BID/full-length BID), indicating activation of the extrinsic apoptotic pathway, while at 24 h, both AβQ22 and Aβ42 promoted BID cleavage. The plot shows % change in tBID/BID compared to Ctrl BVSMCs. For (**A**–**C**), data and images represent the combination of at least 3 independent experiments, each with 2 replicates. Data are plotted as mean + SEM. For (**A**,**B**), statistical significance was evaluated by two-way ANOVA followed by Šídák’s multiple comparisons test. ** = *p* < 0.01, compared to untreated BVSMCs. For (**C**), statistical significance was evaluated by one-way ANOVA followed by Tukey’s multiple comparisons test. ** = *p* < 0.01, *** = *p* < 0.001, **** = *p* < 0.0001, compared to untreated BVSMCs.

Caspase 8 activation is at the crossroads of the extrinsic and intrinsic apoptotic pathways. Once DRs are engaged, caspase 8 dimerizes and is activated by auto-proteolysis, with subsequent release of the cleaved fragment into the cytosol. Active caspase 8 cleaves its substrates, including BID, which triggers the release of CytC from the mitochondria, leading to the activation of caspase 9 through the apoptosome. To investigate whether Aβ challenge induced DR4- and DR5-mediated caspase 8 activation in BVSMCs, we assessed the expression of cleaved caspase 8 at 2, 6, and 24 h by Western blot after AβQ22 and Aβ42 oligomer treatment. As expected, BVSMCs showed active caspase 8 levels to have significantly increased after 24 h of incubation with AβQ22 and Aβ42 oligomers (Figure 5A and Figure 5B, respectively), with trends toward increases at earlier time points. Accordingly, we observed that both AβQ22 and Aβ42 species induced a significant increase in tBID at 24 h, compared to untreated BVSMCs, confirming that caspase 8-mediated apoptotic events occur following Aβ treatment. Interestingly, Bid was cleaved as early as 6 h following Aβ42 treatment in BVSMCs, with no significant early changes after AβQ22 challenge (Figure 5C). Truncated BID (tBID) is known to inhibit anti-apoptotic Bcl2-like proteins and promote MOMP. These events result in CytC release into the cytosol and ultimately lead to the activation of caspase 9 through the intrinsic apoptotic pathway

**Figure 6:**
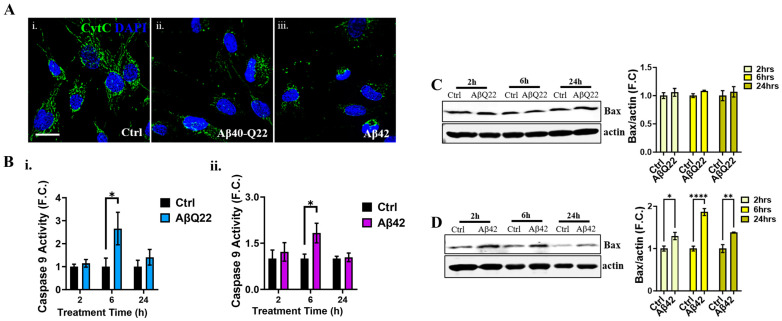
**Aβ oligomers cause Cytochrome C release and caspase 9 activation in BVSMCs.** (**A**) ICC representative images show that, compared to untreated BVSMCs (**i**), 6 h treatment with 10 μM AβQ22 (**ii**) and Aβ42 (**iii**) oligomers triggers loss of Cytochrome C (CytC) mitochondrial signal (green), indicating that Aβ induces the release of CytC from mitochondria to the cytosol, and thus mitochondria-meditated cell death pathway. Cell nuclei are stained with DAPI (blue). Original magnification, 60×. Scale bar, 25 μm. (**B**) Caspase 9 activity was tested at 2, 6, and 24 h, following AβQ22 (**i**) and Aβ42 (**ii**) treatment in BVSMCs. Both AβQ22 (**i**) and Aβ42 (**ii**) significantly induce caspase 9 activation after 6 h challenge, confirming that Aβ induces the intrinsic pathway of apoptosis in BVSMCs. Data are plotted as F.C. of relative Ctrl. (**C**,**D**) WB analysis of pro-apoptotic factor BAX in BVSMCs following 2, 6, and 24 h 10 μM AβQ22 (**C**) and Aβ42 (**D**) challenge**.** AβQ22 (**C**) does not affect Bax expression over time. Conversely, Aβ42 (**D**) triggers significant overexpression of Bax as early as 2 h, which is maintained till 24 h. Graphs show fold change (F.C.) of Bax normalized to actin, and compared to Ctrl. For (**A**–**D**), data and images represent the combination of at least 3 independent experiments, each with 2 replicates. For (**B**–**D**), plots show mean + SEM and statistical significance was evaluated by two-way ANOVA followed by Šídák’s multiple comparisons test. * = *p* < 0.05, ** = *p* < 0.01, and **** = *p* < 0.0001, compared to Ctrl BVSMCs.

We therefore examined CytC release from the mitochondria by immunocytochemistry. Interestingly, BVSMCs already showed a release of CytC from the mitochondria after 6 h treatment with both AβQ22 and Aβ42, as indicated by the reduced CytC staining in mitochondria relative to control cells (which showed a clear chain-like mitochondrial localization of CytC) (Figure 6A). The loss of mitochondrial CytC was accompanied by increased fragmentation of the mitochondrial chains. Once in the cytosol, CytC stimulates the activation of caspase 9 through formation of the apoptosome. As expected, incubation of BVSMCs with both AβQ22 or Aβ42 caused a significant increase in caspase 9 activity at 6 h relative to no-peptide controls (Figure 6B), with no changes at earlier (2 h) or later (24 h) time points. These results suggest that AβQ22 and Aβ42 oligomers induce mitochondria-mediated apoptosis as early as 6 h, leading to CytC release into the cytosol and the subsequent activation of caspase 9, even at time points preceding those of caspase 8 activation (24 h). Thus, intrinsic and extrinsic apoptotic pathways appear to be both independently activated by Aβ challenge in BVSMCs.

Because Bax overexpression is known as one of the initiators of the activation of the intrinsic, mitochondria-mediated, apoptotic pathway, we sought to determine whether Bax expression changed in the presence of Aβ oligomers. While AβQ22 (Figure 6C) treatment did not affect the levels of the pro-apoptotic Bax, Aβ42 oligomers (Figure 6D) significantly increased Bax expression after 2, 6, and 24 h of treatment relative to untreated BVSMCs, suggesting an involvement of different mediators in the initiation of intrinsic apoptosis by the two peptides. Altogether, these data suggest that Aβ42 and AβQ22 oligomers trigger mitochondria-mediated apoptosis and caspase 9 activation, in parallel to DR4/5-mediated extrinsic apoptosis, in BVSMCs.

### 3.5. Carbonic Anhydrase Inhibition Attenuates Caspase 9 Activation and Loss of Mitochondrial ∆Ψ in BVSMCs

**Figure 7:**
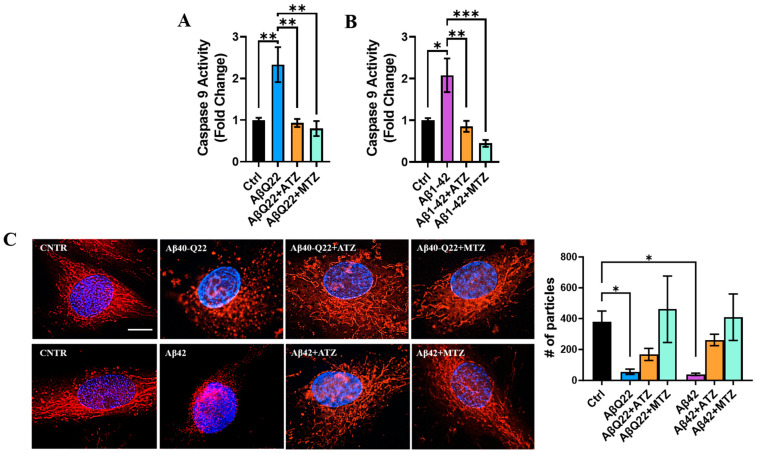
**CAIs reduce Aβ-mediated caspase 9 activation and loss of mitochondrial membrane potential.** (**A**,**B**) Caspase 9 activity was measured in BVSMCs following 6 h Aβ treatment in the presence or absence of CAIs. Both 10 μM AβQ22 (**A**) and Aβ42 (**B**) oligomers significantly increased caspase 9 activity, compared to untreated BVSMCs (Ctrl). ATZ and MTZ significantly rescued AβQ22- (**A**) and Aβ42- (**B**) mediated caspase 9 activation. Graphs represent 3 independent experiments, each with at least 2 replicates, and show F.C. caspase 9 activation compared to Ctrl. Data are plotted as mean + SEM and statistical significance was evaluated by one-way ANOVA followed by Tukey’s multiple comparisons test. * = *p* < 0.05, ** = *p* < 0.01, *** = *p* < 0.001, compared to Ctrl. (**C**) Representative images of MitoTracker CMHX2ROS staining (red). In contrast to CNTR BVSMCs, which show healthy mitochondrial membrane potential (∆*Ψ*), 6 h challenge with 10 µM oligomeric AβQ22 and Aβ42 prompted a reduction in MitoTracker accumulation, corresponding to reduced ∆Ψ. The co-treatment with CAIs (both ATZ and MTZ) rescued the loss of ∆*Ψ*. Cell nuclei are stained with DAPI (blue). Images are representative of 3 independent experiments. Original magnification, 100×. Scale bar, 10 μm. On the right is the relative quantification. Data are plotted as mean + SEM and statistical significance was evaluated by Kruskal–Wallis followed by Dunn’s multiple comparisons test. * = *p* < 0.05.

As described above, oligomeric AβQ22 and Aβ42 trigger CytC release from the mitochondria and subsequent caspase 9 activation in BVSMCs (Figure 6A,B). Our lab has shown that the CAIs acetazolamide (ATZ) and methazolamide (MTZ) prevent apoptosis and the release of CytC in Aβ-treated brain microvascular endothelial, glial, and neuronal cells [7,23,31], in vitro and in vivo [23,24]. Hence, we asked whether CAIs could prevent Aβ oligomer-induced caspase 9 activation in BVSMCs. Here, we show that both CAIs completely reverted caspase 9 activation in BVSMCs challenged with AβQ22 (Figure 7A) and Aβ42 (Figure 7B), suggesting that ATZ and MTZ rescue mitochondria-mediated apoptotic pathways in these mural cells. To evaluate whether this rescue was mediated by a protective effect of CAIs on the loss of BVSMC mitochondrial membrane potential (∆Ψ), we assessed the effects of CAIs on ∆Ψ in BVSMCs treated with Aβ using MitoTracker CMH2XROS (Figure 7C), a dye that only enters the mitochondria in the presence of a healthy membrane potential. We observed that BVSMCs challenged with both oligomeric Aβ peptides, compared to untreated cells, displayed an evident decrease in MitoTracker CMH2XROS signal at 6 h, and—where signal was still detectable—highly fragmented mitochondria, demonstrating that AβQ22 and Aβ42 oligomers severely affect mitochondrial ∆Ψ and mitochondrial structure in BVSMCs. Remarkably, co-treatment with ATZ and MTZ restored mitochondrial ∆Ψ, as well as mitochondrial structure.

To assess if this protection was due to the effects of the CAIs on mitochondrial respiration efficiency, we performed Seahorse analysis in Aβ-treated BVSMCs in the presence of ATZ or MTZ. Treatment with either ATZ or MTZ in the presence of AβQ22 oligomers did not ameliorate mitochondrial OCR, basal respiration, maximal respiration, spare respiratory capacity, and ATP production after 24 h, but resulted in significant improvement in non-mitochondrial oxygen consumption (Supplementary Figure S1A). In Aβ42-treated BVSMCs, CAIs partially rescued maximal respiration (ATZ) and spare respiratory capacity (both ATZ and MTZ) (Supplementary Figure S1B), although no improvement in basal respiration, non-mitochondrial oxygen consumption, or ATP production was observed (Supplementary Figure S1B).

Altogether, these data suggest that CAIs significantly prevent the loss of mitochondrial membrane potential and apoptosis induced by oligomeric Aβ in BVSMCs. In addition, we showed that the effects of CAIs on mitochondrial respiration are peptide-dependent and do not appear to be the primary mechanism responsible for their protective effects.

### 3.6. Carbonic Anhydrase Inhibitors Reduce Caspase 3 Activation in Brain Vascular Smooth Muscle Cells In Vivo in a Mouse Model of CAA

To confirm the beneficial effects of CAIs against BVSMC apoptosis in vivo, we tested whether CAIs reduced Aβ-mediated caspase 3 activation in brain arteries of TgSwDI mice, an in vivo model of cerebrovascular amyloidosis with CAA. TgSwDI mice express human Amyloid-β Precursor Protein (hAPP), carrying the Swedish, Dutch, and Iowa mutations. The model is characterized by the accumulation of Aβ at the brain vasculature, together with diffused parenchymal Aβ deposition and neuroinflammation. The treatment with ATZ or MTZ started when animals were 7/8 months old, when cerebral Aβ accumulation is mild in this model, to mimic clinical treatment in MCI (Mild Cognitive Impairment) patients, as we recently described [24].

**Figure 8:**
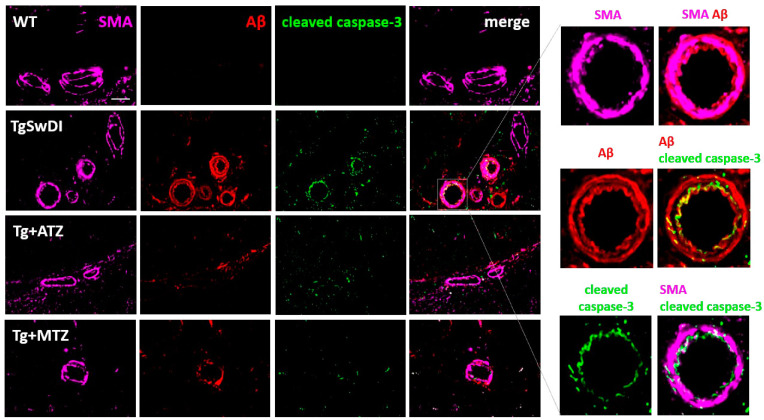
**CAIs reduce Aβ burden and Aβ-driven caspase-3 activation in smooth muscle cells in TgSwDI animals.** Representative immunofluorescence images of leptomeningeal arteries of the hippocampal fissure of 16-month-old TgSwDI mice. Compared to WT, untreated TgSwDI animals exhibited Aβ overload (red) and evident caspase 3 activation (green) in smooth muscle actin (SMA, magenta)-positive cells, which were both decreased by 8 months of CAI treatment. Original magnification, 60×. Scale bar, 25 μm. The magnified images depict the overlap between the signals.

We assessed Aβ accumulation and active caspase 3 in BVSMCs of the leptomeningeal arteries in the hippocampal fissure. We found that 16-month-old Tg mice had a substantial vascular Aβ deposition (Aβ signal colocalizing with Smooth Muscle Actin, SMA, marker of BVSMCs), compared to aged-matched WT mice (Figure 8). Confirming our in vitro results, we detected cleaved (active) caspase 3 colocalizing with Aβ in the BVSMCs of TgSwDI animals. Notably, both CAIs mitigated caspase 3 activation in BVSMCs, as well as BVSMC Aβ deposition, indicating that ATZ and MTZ rescue Aβ-driven SMC apoptosis, while improving Aβ clearance at the arterial walls.

## 4. Discussion

In this study, we demonstrate that both intrinsic (mitochondria-mediated) and extrinsic (DR4/5-mediated) apoptotic pathways are activated in BVSMCs by oligomeric Aβ42 and AβQ22, which are peptides that are toxic for the cerebral vasculature. We also reveal that these oligomeric Aβ species induce mitochondrial metabolic deficits and impair OXPHOS in BVSMCs. Importantly from a therapeutic standpoint, we show that the FDA-approved CAIs MTZ and ATZ prevent these pro-apoptotic effects by reducing the loss of mitochondrial ∆Ψ, CytC release, and caspase 9 activation in BVSMCs in vitro. Of note, treatment with the CAIs also reduces caspase 3 activation and Aβ deposition in BVSMCs in vivo in a mouse model of CAA, the TgSwDI mice.

Different Aβ peptides and aggregates have been shown to induce alternative toxicity pathways in cerebrovascular cells, with oligomers preferentially driving apoptosis in endothelial cells, while fibrillar aggregates more robustly induce BBB permeability [32]. Studies investigating the specific pathways activated by Aβ peptides and aggregates in BVSMCs are lacking and extremely needed, as their understanding can lead to the discovery of novel targets for drug development, which may help reduce the vascular demise in AD and CAA. Importantly, new therapeutic strategies for AD, such as the recently FDA-approved monoclonal anti-Aβ antibodies, induce, as a side effect, the development of ARIA (ARIA-E for edema and ARIA-H for hemorrhage) [12]. There is a recognized relationship of ARIA with CAA and cerebrovascular dysfunction [4,43]. The lack of knowledge and therapeutic strategies available for ARIA is worrisome in view of the clinical application of these new therapies. Understanding the pathways by which BVSMCs, cells lining the blood vessels and participating in the clearance of amyloids [4,5], are destroyed by Aβ challenge, as well as introducing novel possible therapeutic strategies to prevent their demise, which could be given alongside anti-Aβ antibodies or at the early signs of ARIA, is an essential area with very few current research efforts. This study helps in highlighting novel molecular targets and a potential therapeutic strategy against vascular amyloidosis and ARIA.

Reports from our lab and others suggest that BVSMCs undergo apoptosis when challenged with Aβ in AD and CAA models [7,44,45]. However, very little is known about the specific apoptotic pathways activated by Aβ challenge in BVSMCs, and possible therapeutic strategies and targets are lacking.

Our previous studies in cerebral microvascular endothelial cells have pointed out that Aβ functions as an alternative ligand for the death receptors DR4 and DR5, activating the extrinsic apoptotic pathway and leading to caspase 8 activation in these cells [8,46]. Other DRs, such as p75, are also activated by Aβ in neuronal cells, promoting neurotoxicity [47,48,49,50]. However, nothing was known about the effects of Aβ on death receptors DR4- and DR5-mediated apoptosis in BVSMCs. Here, we show that both DR4 and DR5 are upregulated and exposed on the cell membrane at times (6 h and 24 h) well preceding the execution of apoptosis (measured as DNA fragmentation), which is not significant until 48 h after challenge. These results, together with the activation of caspase 8 (at 24 h), indicate that DR4- and DR5-mediated extrinsic apoptotic pathways contribute, at least in part, to the apoptotic outcome in BVSMCs challenged with Aβ oligomers. Indeed, we demonstrated that when DR4 and/or DR5 are silenced, the activation of caspase 3 induced by both AβQ22 and Aβ42 is dramatically reduced.

Interestingly, caspase activation starts in these cells much earlier than DNA fragmentation, which is the final step of the apoptotic machinery. In fact, multiple brain cells including glial cells and neurons have been shown to present caspase 3 activation in the absence, or preceding, complete cell death. Caspase 3 activation modulates mechanisms such as axon guidance, synaptogenesis, and maintenance of synaptic plasticity, including in AD models [51]. It was also hypothesized that caspase 3 activation can be a stress mechanism used by brain cells as a “danger” signal to trigger interaction between various cell types, such as stimulating clearance of specific neuronal synapses by glial cells [52].

Since mitochondrial metabolic changes often precede and accompany apoptosis, we assessed the mitochondrial OXPHOS metabolism preceding the final execution phase of apoptosis (DNA fragmentation, significant at 48 h) by Seahorse analysis after 24 h challenge. We observed a pronounced loss of OCR for all measured respiratory outcomes in the presence of AβQ22 and, even more dramatically, upon Aβ42 treatment. These data suggest that mitochondrial metabolic dysfunction precedes the final execution of apoptosis in Aβ-challenged BVSMCs. Because mitochondrial respiration is essential for cell function and contractile force in cells such as SMCs, our results may also explain the reduced functional and contractile activity of BVSMCs in ADRD (AD and related dementia) pathologies, which impairs vascular fitness, perivascular clearance, and IPAD, and further contributes to Aβ accumulation [4,5,45,53,54,55]. Indeed, mitochondrial dysfunction and reduced mitochondrial respiratory metabolism have been recognized to be among the earliest and causal events in AD pathogenesis [54,56,57,58,59,60,61,62,63,64,65]. However, this is the first report demonstrating that mitochondrial OXPHOS in BVSMCs is impacted by Aβ oligomers. We postulate that this loss of mitochondrial respiration efficiency and ATP production may contribute, even before or in the absence of overt cell death, to the impaired motive force of BVSMCs, likely disrupting both IPAD and glymphatic flow and resulting in the accumulation of Aβ at the brain vasculature, which exacerbates CAA and AD pathology.

During DR-mediated apoptosis, BID is cleaved by active caspase 8 to form tBID, which triggers the involvement of the mitochondria, with the release of CytC and activation of caspase 9 [8]. Interestingly, our study shows that caspase 9 is activated in Aβ-challenged BVSMCs after 6 h treatment. This precedes the activation of caspase 8, suggesting a parallel and independent induction of both intrinsic mitochondria-mediated apoptotic pathways and DR-mediated extrinsic apoptotic pathways. Accordingly, the release of CytC from the mitochondria, necessary for the formation of the apoptosome and caspase 9 activation, is also evident after 6 h treatment. It is known that Aβ oligomers increase the production of ROS, inducing high levels of oxidative stress [23,66], which may explain the early activation of intrinsic apoptosis observed in BVSMCs following oligomeric AβQ22 and Aβ42 treatment. Oxidative damage is also known to promote further Aβ accumulation [67,68], mitochondrial permeability transition pore formation [34,69], CytC release [23,31,70], and overall mitochondrial dysfunction [23,71].

Making a comparison between the toxicity of AβQ22 and Aβ42 oligomers is complex and the effects are cell-dependent. In the present manuscript, it appears that apoptosis, measured as DNA fragmentation (particularly at 72 h) and caspase 3 activation, is slightly more pronounced in BVSMCs treated with Aβ42 oligomers. This is in line with the stronger mitochondrial respiration deficiency induced by this peptide, as well as with the increased levels of Bax, suggesting that Aβ42 activates the intrinsic apoptotic pathway more powerfully than AβQ22 in BVSMCs. Nevertheless, it has also been shown that Bax translocation to the mitochondrial membrane induces the intrinsic apoptotic pathway independently from its overexpression [72], which may explain why we did not observe any significant change in Bax expression with AβQ22 treatment.

Therapeutic strategies to improve BVSMC health in AD and CAA are still lacking. Our lab has recently shown that FDA-approved CAIs are able to decrease cognitive dysfunction and Aβ deposition in a mouse model of AD/CAA by improving cerebrovascular function and reducing inflammation [24]. Here, we tested for the first time on primary BVSMCs in vitro whether MTZ and ATZ were able to reduce mitochondria-mediated apoptotic pathways. In pathological conditions, the perturbation of membrane structure and function can trigger mitochondrial depolarization, contributing to activation of the intrinsic apoptotic cascade, and resulting in caspase 9 activation. Our results showed that both oligomeric Aβ peptides prompt mitochondrial depolarization. Remarkably, co-treatment with ATZ and MTZ rescued this Aβ-mediated loss of ∆Ψ. To further confirm the beneficial effect of CAIs on intrinsic, mitochondria-mediated apoptosis, we tested the activation of caspase 9 and found that ATZ and MTZ reduce active caspase 9 to levels comparable to untreated BVSMCs. These findings demonstrate that CAIs are effective at inhibiting mitochondria-mediated apoptotic pathways induced by Aβ in BVSMCs. This study and others from our group suggest that ROS production, mitochondrial membrane depolarization, and CytC release, key events in AD pathogenesis [23,54,58,65,73], may represent the main targets of ATZ and MTZ. Confirming this, CAIs rescued Aβ-induced apoptosis in BVSMCs by inhibiting the loss of ∆Ψ, CytC release from the mitochondria, and caspase 9 activation. Our data also demonstrate that mitochondrial energy production is reduced in Aβ-challenged BVSMCs, although it is not the primary mechanism targeted by CAIs. Thus, in BVSMCs, the beneficial outcomes of CAIs against oligomeric AβQ22 and Aβ42 peptides are likely due to the preserved mitochondrial membrane potential and reduced CytC release, important contributors to BVSMC apoptotic cell death. In our hands, both amyloid peptides induced the decrease in oxygen consumption and ATP production, without affecting the proton leak across the inner mitochondrial membrane. This OXPHOS reduction likely mediates the loss of mitochondrial membrane potential. Although we did not observe a rescue with ATZ and MTZ in the OCR, CAIs mitigated spare respiratory capacity and non-mitochondrial oxygen consumption, and, more effectively, reversed the loss of mitochondrial membrane potential. This is an interesting finding, in line with our hypothesis that ATZ and MTZ prevent the production of protons within the mitochondrial matrix (through their inhibition of mitochondrial CAs), especially in the presence of stressful stimuli, such as Aβ oligomers. This reduction in positive charge in the mitochondrial matrix compared to the intermembrane space would thereby maintain an effective mitochondrial membrane potential even during challenge, therefore decreasing the downstream toxic consequences, such as CytC release and induction of cell death.

Importantly, we confirmed the protective effects of CAIs on BVSMCs in vivo in TgSwDI animals, a model of CAA. Here, we show that both ATZ and MTZ reduce caspase 3 activation as well as Aβ deposition in the BVSMCs of hippocampal fissure arteries. This suggests that CAIs, by reducing Aβ-driven apoptosis, can ameliorate BVSMC function, facilitating IPAD/clearance mechanisms, and thus decreasing Aβ deposition around the vasculature, BBB permeability, and microhemorrhages in the AD and CAA brain. We previously reported that CAIs rescue apoptotic events in brain endothelial and glial cells in this mouse model of cerebral amyloidosis with CAA [24]. This combined evidence introduces these FDA-approved CAIs as a potential novel pharmacological strategy to prevent vascular demise in AD and CAA, which may also be employed concomitantly with the recent FDA-approved anti-Aβ antibodies, or at the early manifestations of ARIA, to reduce cerebrovascular dysfunction. It is known that 1000 mg ATZ has an acute supramaximal vasodilatory effect [74]. The same concentration is used to prevent high altitude sickness [75] Notably, in our mouse model, CAIs were employed at a concentration (20 mg/kg), which, translated to human doses by allometric scaling, corresponds to 182 mg/day for a 70 kg person, many folds lower than the ones currently clinically used and therefore safe for chronic systemic administration, as we recently described [24].

Although MTZ and ATZ are non-selective-CAIs, both of them have high activity on the mitochondria-specific CA isoforms, CA-VA and -VB [76]. We have recently shown that mitochondrial CA-VB expression is upregulated in TgSwDI brains, and MTZ and ATZ rescue this effect [24]. We have also demonstrated that CA-VB is overexpressed in human brains with CAA or AD+CAA [24], as well as in brain endothelial cells treated with vasculotropic AβQ22 in vitro. Since, in our model, the main detrimental effects observed in BVSMCs are mediated by mitochondrial pathways, mitochondrial CA-VA and -VB may also be the most important mediators of these protective effects in these and other mural cells. More studies exploring these specific targets in BVSMCs or other mural cells, such as pericytes, are needed and currently ongoing in our group.

Altogether, the results of the present study shed light on the specific mechanisms and targets mediating Aβ-driven mitochondria- and DR-induced apoptosis in BVSMCs and provide new valuable data to sustain the development of CAIs as a possible therapeutic strategy for CAA, AD, as well as for ARIA-like complications arising with the recently FDA-approved AD therapies.

## Data Availability

The data included in this article will be shared upon reasonable request to the corresponding author and will be made available after publication in data biorepositories.

## Supplementary Materials

The following supporting information can be downloaded at: https://www.mdpi.com/article/10.3390/cells12242840/s1, Figure S1: Effects of CAIs on mitochondrial respiration in Aβ-treated BVSMCs. (**A**,**B**) Measurement of mitochondrial respiration in BVSMCs, treated for 24 h with 10 μM AβQ22 (**A**) and Aβ42 (**B**)**,** in presence or absence of CAIs. (**A**) AβQ22 significantly impairs oxygen consumption rate (OCR) in non-mitochondrial oxygen consumption (**iii**) and spare respiratory capacity (**iv**) measurements, without affecting basal respiration (**i**), maximal respiration (**ii**), ATP production (**v**), and proton leak (**vi**). Both CAIs rescue non-mitochondrial oxygen consumption (**iii**)**.** (**B**) Aβ42 significantly impairs basal respiration (**i**), maximal respiration (**ii**)**,** non-mitochondrial oxygen consumption (**iii**)**,** spare respiratory capacity (**iv**), and ATP production (**v**) in BVSMCs, with no effect on proton leak (**vi**). For (**A**,**B**), data are plotted following normalization to protein concentration. Graphs represent 3 independent experiments, with 6 replicates per group. Data are plotted as mean + SEM and statistical significance was evaluated by one-way ANOVA followed by Tukey’s multiple comparisons test. * = *p* < 0.05, ** = *p* < 0.01, *** = *p* < 0.001, and **** = *p* < 0.0001, compared to untreated BVSMCs.

## Abbreviations

**Abbreviation**	**Keywords**
Aβ	Amyloid beta
Aβ42	Amyloid beta 1-42
AβQ22	Amyloid beta 1-40, Dutch mutation
CAA	Cerebral Amyloid Angiopathy
AD	Alzheimer’s Disease
BVSMCs	Brain Vascular Smooth Muscle Cells
TRAILs	TNF-Related Apoptosis-Inducing Ligands
DRs	Death Receptors
IPAD	Intramural Peri-Arterial Drainage
ARIA	Amyloid-Related Imaging Abnormalities
FADD	Fas-Associated protein with Death Domain
DISC	Death-Inducing Signaling Complex
BID	BH3-Interacting Domain death agonist
MOMP	Mitochondrial Outer Membrane Permeabilization
CytC	Cytochrome C
ROS	Reactive Oxygen Species
CAs	Carbonic Anhydrases
MTZ	Methazolamide
ATZ	Acetazolamide
OCR	Oxygen Consumption Rate
hAPP	Human Amyloid beta Precursor Protein
ADRD	AD and related dementia
