# Supplementary material for: Amyloid Beta Oligomers Activate Death Receptors and Mitochondria-Mediated Apoptotic Pathways in Cerebral Vascular Smooth Muscle Cells; Protective Effects of Carbonic Anhydrase Inhibitors
Source: Cells. 2023 Dec 14;12(24):2840. doi: 10.3390/cells12242840 (PMC10741628; doi:10.3390/cells12242840)
Supplement: Supplementary file 1 [file cells-12-02840-s001.zip › cells-2740160-supplementary.pdf]

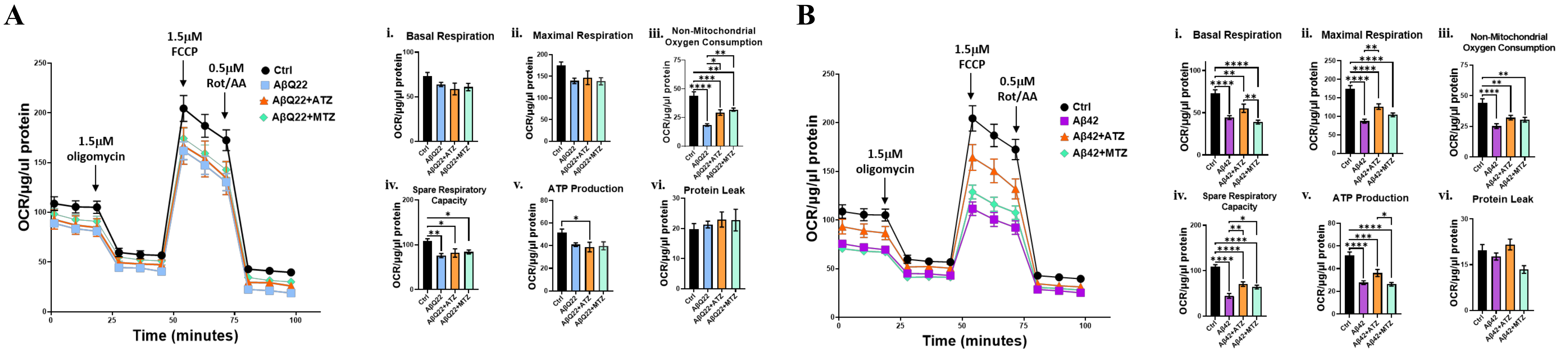

Figure S1. Effects of CAIs on mitochondrial respiration in Aβ-treated BVSMCs.

Supplementary Figure Legends

Figure S1.

Effects of CAIs on mitochondrial respiration in Aβ-treated BVSMCs.

A) and **(B)** Measurement of mitochondrial respiration in BVSMCs, treated for 24hrs with 10μM Aβ40-Q22 **(A)** and Aβ42 **(B),** in presence or absence of CAIs. **A)** Aβ40-Q22 significantly impairs oxygen consumption rate (OCR) in non-mitochondrial oxygen consumption **(iii)** and spare respiratory capacity **(iv)** measurements, without affecting basal respiration **(i)**, maximal respiration **(ii)**, ATP production **(v)** and proton leak **(vi)**. Both CAIs rescue non-mitochondrial oxygen consumption **(iii). B)** Aβ42 significantly impair basal respiration **(i)**, maximal respiration **(ii),** non-mitochondrial oxygen consumption **(iii),** spare respiratory capacity **(iv)**, and ATP production **(v)** in BVSMCs, with no effect on proton leak **(vi)**. For **(A)** and **(B)**, data are plotted following normalization to protein concentration. Graphs represent 3 independent experiments, with 6 replicates per group. Data are plotted as mean + SEM, and statistical significance was evaluated by One-way ANOVA, followed by Tukey's multiple comparisons test. *= $p<0.05$, **= $p<0.01$, ***= $p<0.001$, and ****= $p<0.0001$, compared to untreated BVSMCs**.**
